# Boosting Hydroformylation via Reactant Enrichment in Covalent Triazine Frameworks with Atomically Dispersed Rh

**DOI:** 10.3390/ma18122691

**Published:** 2025-06-07

**Authors:** Xinguo Li, Xiangjie Zhang, Gaolei Qin, Peng He, Yajuan Hao

**Affiliations:** 1 School of Chemistry and Chemical Engineering, Shanxi University, Taiyuan 030006, China; lixinguo0105@163.com; 2 State Key Laboratory of Coal Conversion, Institute of Coal Chemistry, Chinese Academy of Sciences, Taiyuan 030001, China; zhangxiangjie18@mails.ucas.ac.cn (X.Z.); qingaolei21@mails.ucas.ac.cn (G.Q.)

**Keywords:** heterogeneous catalyst, covalent triazine frameworks, hydroformylation, long chain olefin

## Abstract

Hydroformylation is one of the most widely applied homogeneous catalytic processes in the chemical industry, constituting the predominant manufacturing platform for aldehyde synthesis at commercial scales. Nevertheless, hydroformylation shares with traditional homogeneous catalysis the inherent limitation of difficult catalyst recovery and recycling. Developing heterogeneous catalysts for such reactions is thus critically needed. Herein, a stable nitrogen-rich covalent triazine framework (CTF) was synthesized via a mild Friedel–Crafts alkylation method and employed as a support for Rh single-atom catalysts (Rh/CTF-TPA). In the hydroformylation of 1-decene, the Rh/CTF-TPA catalyst exhibits an exceptional reaction efficiency (TOF > 1900 h^−1^), outperforming the homogeneous Rh(CO)_2_(acac). Experimental and characterization results revealed that the CTF support enhances catalytic performance through two key mechanisms: (1) strong enrichment of reactants within its special structure, and (2) efficient dispersion of Rh single-atom sites stabilized by abundant nitrogen coordination. This work demonstrates a rational design strategy for heterogeneous hydroformylation catalysts by leveraging nitrogen-rich porous frameworks to synergistically optimize metal anchoring and reactant enrichment, offering a promising alternative to conventional homogeneous systems.

## 1. Introduction

Aldehydes, as pivotal synthetic intermediates, can be further transformed into versatile alcohols, acids, acetals, and other fine chemicals through hydrogenation, oxidation, aldol condensation, reductive amination, and Wittig reactions, demonstrating extensive application value in industrial production, pharmaceutical chemistry, and daily life [1,2,3,4]. Among various synthetic approaches, the hydroformylation of olefins is recognized as one of the most important and atom-economic methods for aldehyde production, with a global annual capacity exceeding 12 million tons [5]. Early hydroformylation processes predominantly employed cobalt-based catalysts, which required harsh reaction conditions such as high syngas pressure to maintain the stability of active species. Subsequent studies have revealed that rhodium catalysts exhibit activity hundreds of times higher than their cobalt counterparts, leading to their current dominance in over 75% of hydroformylation processes [6,7]. Although Rh-based homogeneous catalytic systems offer advantages in activity and selectivity, they suffer from inherent limitations in catalyst–product separation and recyclability.

Heterogeneous catalysis for hydroformylation, achieved by immobilizing active metals (such as Rh, Co, Ru) on solid supports, enables the efficient separation and recycling of catalysts from reaction products [8,9,10]. Compared with homogeneous systems, heterogeneous catalysts can be recovered through simple physical methods such as filtration or centrifugation after the reaction, significantly reducing the loss of precious metals and enhancing economic efficiency. POPs represent a class of crystalline materials characterized by ordered porous architectures constructed from light elements (e.g., C, O, N, B) interconnected via covalent bonds [11]. These materials can be synthesized through relatively simple organic reactions and exhibit outstanding potential in heterogeneous catalysis [12,13,14]. Based on the chemical bonding motifs and building blocks, porous organic polymers (POPs) can be classified into the following categories: covalent triazine frameworks (CTFs), covalent organic frameworks (COFs), polymers of intrinsic microporosity (PIMs), porous aromatic frameworks (PAFs), hyper-crosslinked and conjugated microporous polymers (HCPs and CMPs), carbazole-based porous organic polymers (CPOPs), and porous imine-linked networks (PINs) [15]. Among diverse POPs, CTFs have emerged as particularly promising candidates for catalytic applications due to their fully conjugated structures, high specific surface areas, exceptional thermal/chemical stability [16]. The high surface area and abundant porosity of CTFs ensure excellent accessibility to active sites, facilitating efficient diffusion of gaseous reactants and substrate molecules to catalytic centers, thereby enhancing reaction kinetics [17]. Furthermore, the nitrogen-rich framework enables strong coordination with metal precursors, allowing for precise control over active site configurations. This structural precision serves as a critical factor for achieving regioselective hydroformylation of long-chain olefins to linear aldehydes, demonstrating the material’s unique advantages in catalysis engineering.

Various strategies have been developed to efficiently incorporate metallic species into the porous channels of polymers [18], including in situ synthesis [19] by introducing metal precursors during polymerization, ion exchange [20] relying on pre-functionalized coordination groups, and chemical vapor deposition (CVD) [21] that enables uniform deposition of metal nanoparticles within pores yet remains limited by the thermal stability of polymers. In comparison, the impregnation method has been extensively explored due to its operational simplicity and cost-effectiveness. By modulating precursor concentration and immersion duration, this approach allows for precise control over metal loading content and nanoparticle size, while demonstrating broad compatibility with diverse metal salts and polymer supports. Such versatility positions impregnation as a universally adaptable technique for fabricating supported heterogeneous catalyst.

The synthesis of CTFs via direct triazine ring-forming reactions encompasses both high-temperature methods (e.g., ZnCl₂-catalyzed ionothermal synthesis [22] and P₂O₅-mediated polymerization [23]) and non-high-temperature approaches (e.g., superacid-catalyzed protocols [24] and amidine-based polycondensation [25]). These polymerization strategies enable the construction of well-defined triazine architectures with structural flexibility and scalability. However, high-temperature methods demand harsh reaction conditions, requiring specialized high-temperature-resistant equipment and thermally stable monomers [26], whereas low-temperature routes often rely on tailored catalytic systems or prolonged reaction durations to achieve comparable structural fidelity [27]. Therefore, polymerization strategies utilizing triazine-containing monomers to construct structurally tunable CTFs through relatively straightforward cross-coupling reactions have been actively investigated [28,29]. Pan et al. [30] synthesized high-surface-area nanoporous organic polymers based on 1,3,5-triazine units via the Friedel–Crafts reaction between 2,4,6-trichloro-1,3,5-triazine and tetrahedral structural building blocks. The resulting materials exhibited notable hydrogen uptake capacity and CO_2_-selective adsorption performance, demonstrating their potential for applications in gas adsorption and separation. This synthetic approach offers mild reaction conditions and operational simplicity, where both the monomers and catalysts are inexpensive and readily available, allowing for straightforward implementation in laboratory environments.

Herein, we designed and synthesized a stable and cost-effective CTF material enriched with nitrogen coordination sites, which served as a support for anchoring rhodium species to construct a heterogeneous Rh-based CTF catalyst for long-chain olefin hydroformylation. The rigid conjugated structure and abundant triazine-based nitrogen units of CTFs endow exceptional coordination capability to immobilize Rh active centers effectively. Furthermore, the well-defined porous architecture facilitates the adsorption of reactants, thereby enhancing hydroformylation activity. When evaluated in the probe reaction of 1-decene hydroformylation, the heterogeneous catalyst demonstrated superior catalytic activity and higher linear/branched aldehyde ratio compared to its homogeneous counterpart Rh(CO)_2_(acac). Detailed characterization revealed that the Rh species, coordinated with the nitrogen-rich sites, existed as isolated single-atom sites distributed uniformly on the CTF support. The distinctive architecture of CTF supports endows them with a pronounced capability for olefin enrichment, thereby significantly enhancing the local concentration of reactants at catalytically active sites and boosting hydroformylation activity. This work provides fundamental insights into the rational design of advanced heterogeneous catalysts through synergistic integration of coordination engineering and confinement effects in porous organic frameworks.

## 2. Materials and Methods

### 2.1. Chemicals

Cyanuric chloride (CC, 99%), o-dichlorobenzene (99%), and methanesulfonic acid (99%) were purchased from Aladdin Reagent Co., Ltd. (Shanghai, China). Triphenylamine (TPA, 98%), methanol (AR grade), and tetrahydrofuran (THF, AR grade) were obtained from Innochem Reagent Co., Ltd. (Beijing, China). Acetone (AR grade) and toluene (AR grade) were supplied by Tongguang Reagent Co., Ltd. (Beijing, China). Rh(CO)_2_(acac) (98%, 39% Rh content) was procured from Bide Pharmatech Ltd. (Shanghai, China), and 1-decene (95%) was acquired from Macklin Reagent Co., Ltd. (Shanghai, China).

### 2.2. Synthesis of CTF-TPA and Rh/CTF-TPA

CTF-TPA was synthesized via a mild liquid-phase acid-catalyzed Friedel–Crafts alkylation reaction using CC as the triazine donor. In a 250 mL round-bottom flask, a mixture of TPA (5 mmol) and CC (5 mmol) was dissolved in 150 mL of o-dichlorobenzene. Methanesulfonic acid (70.0 mmol) was added, and the reaction mixture was refluxed at 140 °C for 24 h. The resulting precipitate was isolated from the hot solution by filtration, followed by Soxhlet extraction with anhydrous methanol, THF, and acetone for 12 h to remove unreacted monomers. The target product was obtained as a yellowish-brown flocculent solid after vacuum-drying at 80 °C for 6 h, denoted as CTF-TPA.

Rh/CTF-TPA was prepared via the impregnation method. First, 15 mg of Rh(CO)_2_(acac) was dissolved in 30 mL of toluene to prepare the rhodium precursor solution. Subsequently, 0.5 g of CTF-TPA was dispersed in the precursor solution and stirred at 600 rpm for 12 h. The material was then filtered, washed thoroughly with anhydrous ethanol, and dried in an oven at 80 °C for 6 h to obtain Rh/CTF-TPA. The Rh content was determined to be 1 wt.% by inductively coupled plasma (ICP) analysis.

### 2.3. Characterizations

The FT-IR test was performed using a Bruker VERTEX 70 infrared spectrometer (Karlsruhe, Germany). X-ray diffraction (XRD) analysis was performed using a Bruker D8 Advance diffractometer (Karlsruhe, Germany) with Cu Kα radiation (λ = 0.15406 nm) as the X-ray source, scanning from 5° to 60° at a step size of 0.02°. Thermal stability was evaluated through thermogravimetric analysis (TGA) conducted on a Mettler Toledo TGA/DSC-1 thermal analyzer (Columbus, OH, USA). Morphological characterization was carried out using a FEI Quanta 400 field-emission scanning electron microscope (FESEM) (Hillsboro, OR, USA). Transmission electron microscopy (TEM) observations were performed on a Talos F200A microscope (Thermo Fisher Scientific Brno s.r.o., Brno, Czech Republic) operated at 200 kV. The solid-state UV–Vis diffuse reflectance spectra (DRS) of the polymer powder samples were recorded at room temperature using a UV–Vis spectrophotometer, with barium sulfate (BaSO_4_) employed as the reference standard. X-ray photoelectron spectroscopy (XPS) measurements were acquired on a Thermo Scientific K-Alpha spectrometer (Waltham, MA, USA) employing monochromatic Al Kα X-ray radiation to investigate the chemical states of active metals. Nitrogen adsorption–desorption isotherms were measured at 77 K using a Micromeritics ASAP 2420 analyzer (Norcross, GA, USA), through which the specific surface area, pore size distribution, and micro/mesopore volume of the samples were determined. The pretreated sample (vacuum-dried at 100 °C) was loaded into the sample cell of a PE DSC 7 instrument (PerkinElmer, Waltham, MA, USA) for adsorption enthalpy measurements. The system was initially purged with nitrogen (50 mL/min flow rate) at 298 K for 30 min to stabilize the baseline, followed by switching to propylene gas flow for adsorption. The adsorption enthalpy data were obtained through real-time monitoring of DSC signal variations during the gas adsorption process.

### 2.4. Catalytic Reactions

The catalytic performance of the prepared catalyst, including its activity and selectivity was evaluated through the hydroformylation of 1-decene. Catalyst testing was performed in a 50 mL high-pressure autoclave. Typically, 4 mL of solvent, 15 mg of catalyst, and 5 mmol of substrate were loaded into the autoclave. The reactor was sealed and purged three times with CO (1 MPa), then pressurized to 4 MPa with syngas (H_2_/CO = 1:1). The system was heated to the target reaction temperature under continuous stirring. Upon reaction completion, the reactor was cooled in an ice-water bath for 0.5 h before depressurization. Approximately 1.5 mL of the product was transferred to a chromatography vial for analysis using gas chromatography (GC).

## 3. Results

### 3.1. Catalysts Characterizations

**Scheme 1:**
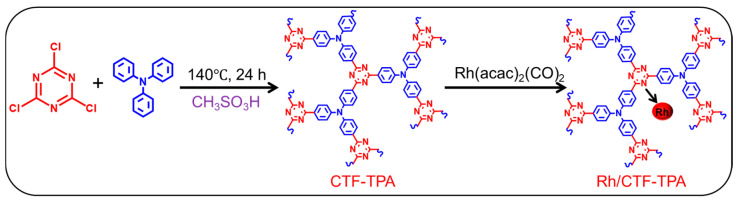
Synthesis of CTF-TPA via a liquid-phase Friedel–Crafts alkylation reaction of cyanuric chloride with triphenylamine catalyzed by methanesulfonic acid, followed by the preparation of Rh/CTF-TPA catalyst through rhodium loading.

According to Scheme 1, the CTF-TPA support was synthesized via a mild Friedel–Crafts alkylation method, followed by the preparation of the Rh/CTF-TPA catalyst through an impregnation approach, with subsequent comprehensive structural and performance characterization. XRD analysis was performed to determine the phase composition of the materials, as shown in Figure S2. CTF-TPA exhibited a broad diffuse peak at 15~25°, characteristic of an amorphous structure. Similar diffraction patterns were observed for Rh/CTF-TPA, confirming that the impregnation process did not alter the structural integrity of the CTF framework. Notably, no discernible diffraction peaks corresponding to metallic Rh were detected at 2θ = 41.0° in Rh/CTF-TPA, suggesting the homogeneous dispersion of Rh species on the CTF support [31].

The microscopic morphology of the catalyst was characterized by SEM, as shown in Figure S3. Low-magnification SEM images revealed that the CTF-TPA material exhibits a complex network structure composed of interwoven micrometer-scale fibers. High-magnification SEM images further revealed tubular through-pores, indicating abundant porosity within the material. This unique morphology significantly enhances the specific surface area of the material. Furthermore, no significant morphological changes were observed in CTF-TPA after Rh loading.

**Figure 1:**
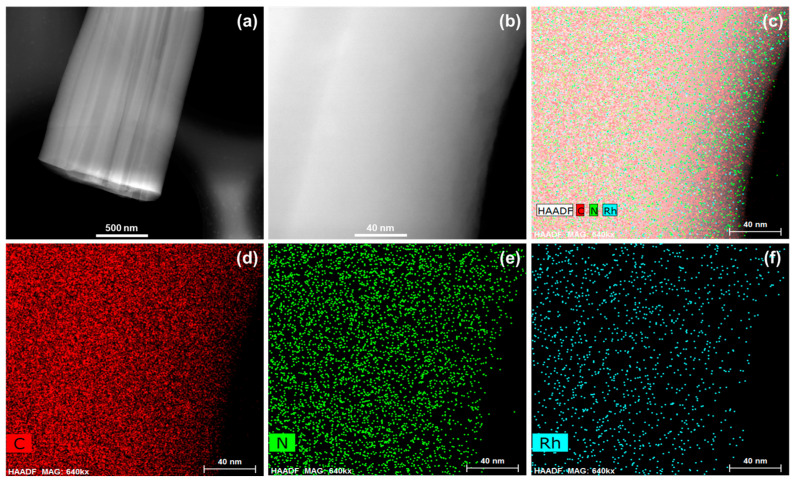
(**a**,**b**) TEM micrographs and (**c**–**f**) elemental mapping profiles of Rh/CTF-TPA catalyst.

The morphology and Rh distribution of Rh/CTF-TPA were investigated by TEM (Figure 1 and Figure S4). As shown in Figure 1a, Rh/CTF-TPA exhibits a well-defined hollow tubular morphology, consistent with SEM observations. Remarkably, no discernible aggregation of Rh nanoparticles was observed in the high-magnification TEM image (Figure 1b) [32]. STEM-EDS elemental mapping analysis (Figure 1c–f) further confirmed the atomic-level homogeneous dispersion of Rh species across the support surface without apparent agglomeration, demonstrating the effective dispersion of Rh active sites through strong metal–support interactions.

Figure S5 presents the N₂ adsorption–desorption isotherms of CTF-TPA and Rh/CTF-TPA. Both CTF-TPA and Rh/CTF-TPA exhibit combined type I and IV adsorption–desorption isotherms with distinct hysteresis loops, indicating their micro/mesoporous structures [33]. The non-closure of the N_2_ adsorption–desorption isotherm curves may arise from the swelling of flexible pore walls in amorphous materials during the high-pressure adsorption process, which fail to fully recover their original structure upon desorption, as well as irreversible desorption behavior induced by unique pore morphology [34,35,36,37]. Table S1 summarizes the Brunauer–Emmett–Teller (BET) surface areas, average pore diameters, and pore volumes of CTF-TPA and Rh/CTF-TPA. The Brunauer–Emmett–Teller (BET) specific surface area of the CTF-TPA material was determined to be 893.6 m^2^ g^−1^. CTF-TPA exhibited predominantly mesoporous structures (as revealed by TEM with tubular/near-cylindrical channels), consistent with the cylindrical pore assumptions of the Barrett–Joyner–Halenda (BJH) model. The pore size distribution curve calculated by the BJH method revealed an average pore diameter of 5.65 nm and a pore volume of 0.65 cm^3^ g^−1^. The presence of a minor fraction of macropores in the pore size distribution analysis may be attributed to interconnected or branched channels formed by tubular pores under fluctuating synthesis conditions. The high specific surface area and abundant porosity of the material not only provide sufficient accommodation space for metallic active centers but also facilitate the molecular diffusion of the substrate, thus enhancing catalytic activity [38]. Compared with the pristine support, the Rh/CTF-TPA catalyst after Rh loading exhibited a decrease in external surface area (S_Ext_) from 368.9 m^2^ g^−1^ to 335.5 m^2^ g^−1^ and a reduction in micropore volume from 0.25 cm^3^ g^−1^ to 0.23 cm^3^ g^−1^, as determined by the t-plot method. These changes suggest that the active Rh species are likely partially dispersed on the carrier surface while simultaneously inducing fractional blockage of micropores. The Rh/CTF-TPA catalyst effectively inherited the structural advantages of the CTF support, confirming the successful implementation of the designed preparation strategy. The uniform dispersion and stable immobilization of Rh species on the carrier establish a robust structural foundation for subsequent catalytic reactions.

TGA was performed under a N_2_ atmosphere for CC, TPA, CTF-TPA, and Rh/CTF-TPA at a heating rate of 10 °C min⁻^1^ over a temperature range of 30~800 °C. As illustrated in Figure S6, both CTF-TPA and Rh/CTF-TPA exhibited excellent thermal stability. The precursor monomers CC and TPA began to decompose at 80 °C and 140 °C respectively, and were completely decomposed around 170 °C and 280 °C. In contrast, the polymeric CTF-TPA displayed significantly enhanced thermal stability, with decomposition initiating at approximately 560 °C. The initial mass loss below 100 °C was attributed to the evaporation of residual moisture, while the slight mass loss between 100 °C and 560 °C originated from the thermal decomposition of unreacted monomers. Remarkably, CTF-TPA retained 63.1% of its mass at 800 °C, whereas Rh/CTF-TPA showed even higher stability with only 24.7% mass loss at the same temperature. This superior thermal stability is advantageous for the application of this catalyst in heterogeneous hydroformylation reactions.

**Figure 2:**
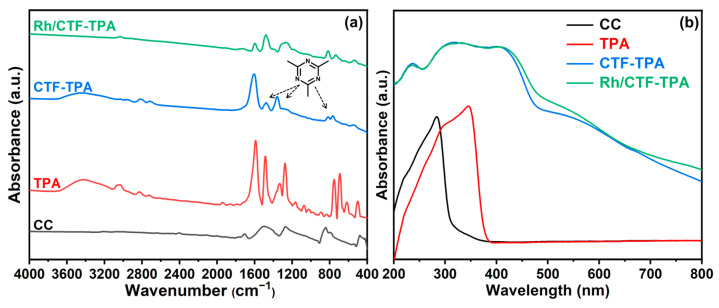
(**a**) FT-IR spectra and (**b**) diffuse reflectance UV−visible spectra of CC, TPA, CTF-TPA, and Rh/CTF-TPA.

The detailed comparison of FT-IR spectra for precursor CC, TPA, CTF-TPA support, and Rh/CTF-TPA catalyst is presented in Figure 2a. In the FT-IR spectrum of CTF-TPA, the characteristic peaks at approximately 1360 cm^−1^ and 1478 cm^−1^ correspond to the stretching vibrations of C-N and C = N bonds in the 1,3,5-triazine rings [39], respectively, showing excellent agreement with the stretching vibration peaks of monomer CC. Compared to CC, the strong C-Cl stretching vibration at 842 cm^−1^ in CTF-TPA virtually disappeared, indicating a nearly complete conversion of C-Cl bonds [40]. In contrast to TPA, CTF-TPA exhibited a newly emerged C-H bending vibration peak at 815 cm^−1^ characteristic of 1,4-disubstituted benzene, while the C-H bending vibration peaks (680~750 cm^−1^ range) associated with monosubstituted benzene disappeared, demonstrating para-directing effects and confirming the occurrence of electrophilic substitution via Friedel–Crafts alkylation between CC and TPA. Furthermore, the close resemblance between the FT-IR spectra of CTF-TPA and Rh/CTF-TPA confirms that Rh incorporation did not alter the structural integrity of CTF-TPA. These findings collectively validate the successful synthesis of both CTF-TPA and Rh/CTF-TPA.

The characterization of the materials via UV–Vis diffuse reflectance spectroscopy provides optical evidence for the successful construction of CTFs through Friedel–Crafts alkylation between CC and TPA. As illustrated in Figure 2b, the synthesized CTF material exhibits a remarkably intensified broad absorption band centered at approximately 400 nm, which is absent in the individual spectra of CC and TPA monomers. This phenomenon can be attributed to the extended π-conjugated system formed through covalent bonding between electron-rich TPA units and electron-deficient triazine rings during the Friedel–Crafts reaction [41,42]. The pronounced bathochromic shift observed in the absorption band relative to the characteristic absorption wavelengths of the monomers (approximately 343 nm for TPA) directly reflects the strong charge transfer interactions between the electron-donating TPA units and the electron-accepting triazine rings within the polymer framework, confirming the successful construction of the conjugated microstructure.

**Figure 3:**
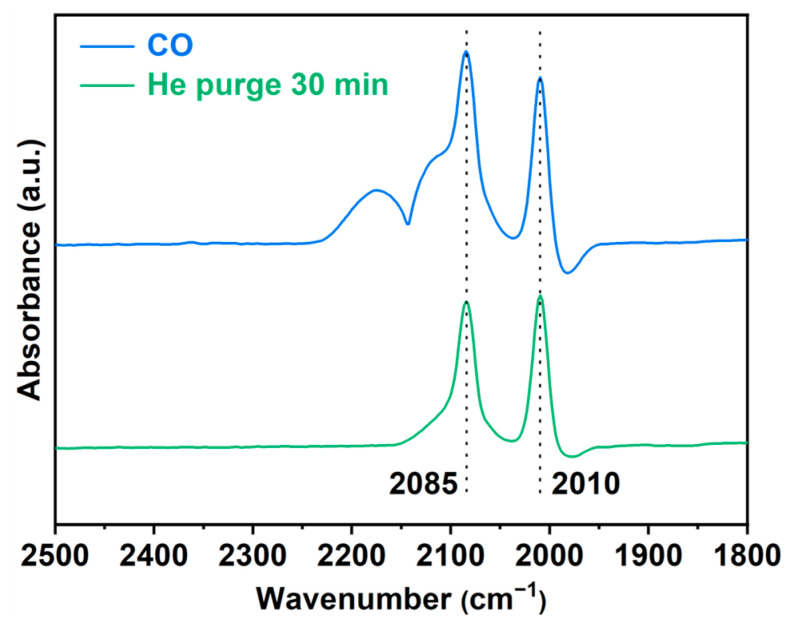
In situ IR spectra of Rh/CTF-TPA upon CO adsorption.

As shown in Figure 3, the CO adsorption FT-IR spectrum of Rh/CTF-TPA reveals two distinct peaks at 2085 cm^−1^ and 2010 cm^−1^ after He purging for 30 min to remove weakly adsorbed CO species [43]. These peaks are attributed to the symmetric and asymmetric stretching vibrations of gem-dicarbonyl species formed by CO adsorption on the active Rh metal sites, providing direct evidence for the single-atom distribution of Rh on CTF-TPA. Notably, no absorption peaks were detected in the 2050~2070 cm^−1^ range typically characteristic of linear CO adsorption on Rh nanoparticles [44].

**Figure 4:**
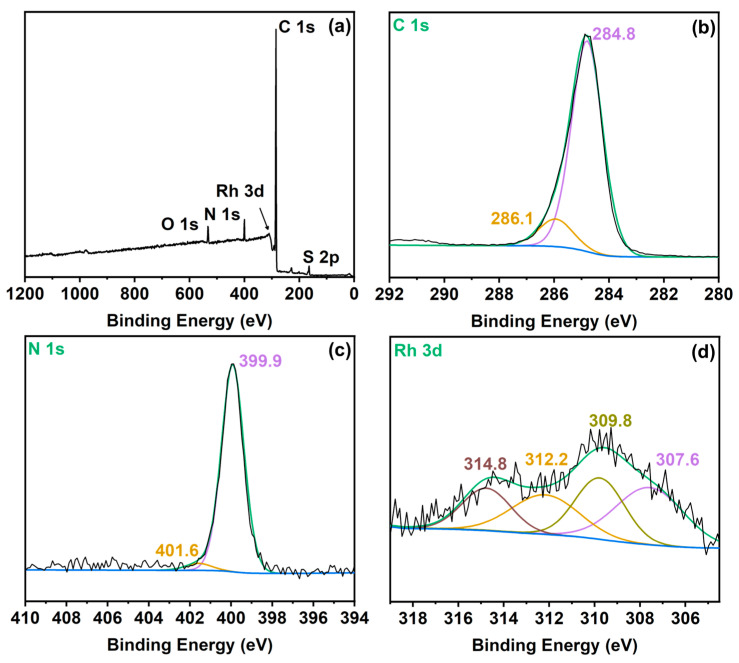
XPS spectra of (**a**) survey; (**b**) C 1s; (**c**) N 1s; (**d**) Rh 3d of Rh/CTF-TPA.

The chemical environment of elements in the synthesized Rh-based covalent triazine framework catalyst was analyzed by X-ray photoelectron spectroscopy (XPS). The XPS survey spectrum (Figure 4a) reveals the presence of C, N, and Rh as primary constituents, with trace amounts of S and O attributed to residual methanesulfonic acid from incomplete removal during catalyst washing—a common phenomenon in acid-catalyzed CTF synthesis protocols. The triazine ring structure exhibits high stability, rendering it resistant to decomposition during this process. Figure 4b shows the C 1s spectrum of Rh/CTF-TPA, where the peak at 284.8 eV is assigned to the C 1s of aromatic ring -C=C- units, and the peak at 286.1 eV corresponds to the C 1s of -C=N- in the triazine ring [45]. As shown in the Rh 3d spectrum of Rh/CTF-TPA catalyst (Figure 4d), the Rh 3d_5/2_ and Rh 3d_3/2_ binding energies were observed at approximately 307.6 eV and 312.2 eV, respectively, which are lower than those of Rh(CO)_2_(acac) (309.4 eV and 314.0 eV, Figure S7a). This energy shift confirms the successful coordination between Rh(CO)_2_(acac) and nitrogen species in the CTF-TPA support. Additionally, the peaks located at 309.8 eV and 314.8 eV can be assigned to Rh^3+^ 3d_5/2_ and Rh^3+^ 3d_3/2_, respectively. The appearance of Rh^3+^ species on the catalyst surface should be ascribed to the oxidation of Rh^+^ species during the catalyst preparation process [46]. The N 1s binding energy of Rh/CTF-TPA catalyst (399.9 eV, Figure 4c) exhibited a notable positive shift compared to the pristine CTF-TPA support (398.8 eV, Figure S7b). These XPS results collectively and unambiguously support the existence of strong electronic interactions between nitrogen coordination sites in the CTF-TPA framework and Rh active species.

### 3.2. Catalytic Performance

**Figure 5:**
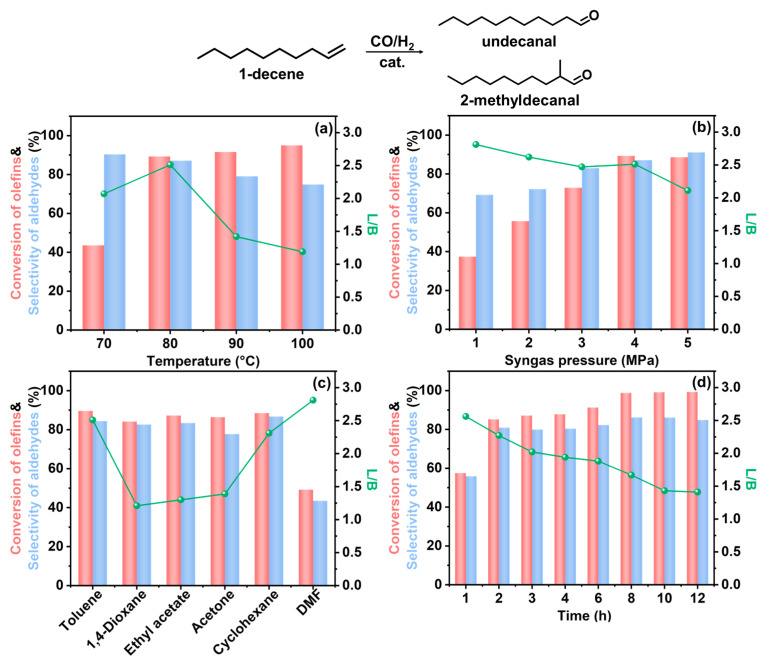
(**a**) Rh/CTF-TPA-catalyzed 1-decene hydroformylation at different temperature; (**b**) Rh/CTF-TPA-catalyzed 1-decene hydroformylation at different syngas pressures; (**c**) Rh/CTF-TPA-catalyzed 1-decene hydroformylation in different solvents; (**d**) effect of reaction time on the Rh/CTF-TPA-catalyzed hydroformylation of 1-decene. Reaction conditions: catalyst (15 mg), 1-decene (5 mmol), solvent (4 mL), T = 80 °C, P(CO/H_2_ = 1) = 4 MPa, t = 2 h. Conversion of olefins and selectivity of aldehydes are represented by red and blue bars, respectively, while the linear/branched aldehyde ratio (L/B) is denoted by circular markers.

To investigate the catalytic activity of Rh/CTF-TPA, the hydroformylation of 1-decene was employed as a probe reaction. A series of experiments were systematically conducted under various reaction conditions to examine the effects of solvent, temperature, and syngas pressure on both catalytic performance and product selectivity. First, the effects of reaction temperature on conversion and selectivity were investigated (Figure 5a, Table S2, Entries 1–4). At 70 °C, the selectivity for undecanal was approximately 90%, with a small amount of internal olefins being produced. However, the conversion of 1-decene remained relatively low (only 43.6% after 2 h). Increasing the reaction temperature significantly enhanced substrate conversion, achieving 89.3% at 80 °C and further improving to 95% at 100 °C. Nevertheless, the linear-to-branched (L/B) ratio of undecanal progressively decreased from 2.46 at 80 °C to 1.19 at 100 °C. This phenomenon originates from the intrinsically lower activation energy characteristic of the linear aldehyde formation pathway. At reduced temperatures, reactant molecules preferentially follow the reaction pathway with lower energy barriers, favoring linear aldehyde production. Furthermore, elevated temperatures intensified 1-decene isomerization, enabling the conversion of internal olefins to branched aldehydes, thereby progressively diminishing the L/B ratio. Subsequently, we systematically investigated the influence of syngas pressure on the reaction performance (Figure 5b, Table S2, Entry 2 and Entries 5–8). The conversion of 1-decene exhibited a gradual enhancement with increasing syngas pressure, reaching comparable levels at 4 MPa and 5 MPa. A slight reduction in the L/B ratio of undecanal was observed with elevated syngas pressures, indicating preferential formation of linear aldehydes under lower pressure conditions. Through comprehensive evaluation of catalytic activity, aldehyde selectivity, and L/B ratio optimization, we conclude that the optimal reaction parameters for Rh/CTF-TPA-catalyzed hydroformylation of 1-decene are 80 °C with 4 MPa syngas pressure.

Furthermore, the solvent was found to significantly influence the catalytic performance (Figure 5c, Table S3). When 1,4-dioxane, ethyl acetate, or acetone were employed as solvents, all systems achieved over 80% conversion of 1-decene but exhibited unsatisfactory linear aldehyde selectivity. While the use of DMF as a solvent resulted in a relatively higher linear-to-branched ratio (L/B = 2.81), the 1-decene conversion decreased substantially to 49.2%. Notably, the Rh/CTF-TPA catalyst demonstrated optimal performance in toluene solvent (Entry 1), achieving 89.6% 1-decene conversion with 84.4% aldehyde selectivity and an L/B ratio of 2.51 for undecanal. Comparable catalytic activity and linear aldehyde selectivity were observed when cyclohexane was used as a solvent. This solvent-dependent behavior can be attributed to the strong solvation effects of highly polar DMF, which weakens olefin adsorption on the catalyst surface and consequently reduces reaction rates. In contrast, low-polarity solvents like toluene facilitate enhanced 1-decene diffusion and adsorption through superior olefin solubility. These findings collectively suggest that both toluene and cyclohexane serve as effective solvents for Rh/CTF-TPA-catalyzed hydroformylation reactions.

Additionally, the influence of reaction time (1–12 h) on the hydroformylation performance was investigated under conditions of 80 °C, 4 MPa syngas pressure, and toluene solvent, as illustrated in Figure 5d. The conversion of 1-decene gradually increased with prolonged reaction time, while the linear/branched aldehyde ratio progressively decreased. This phenomenon is attributed to the gradual participation of byproducts (2-decene and 3-decene) in hydroformylation at extended durations, leading to the formation of branched aldehydes and consequently reduced regioselectivity of the reaction.

Under optimal reaction conditions, a direct comparison between the Rh/CTF-TPA catalyst and the homogeneous Rh(CO)_2_(acac) in 1-decene hydroformylation revealed superior catalytic activity and slightly higher L/B for Rh/CTF-TPA at identical substrate-to-rhodium (S/Rh) ratios (Table 1), achieving a TOF of 1929 h^−1^. Furthermore, compared to the reported Co-based [47,48,49] and Rh-based [50,51,52,53] heterogeneous catalyst systems (as summarized in Table S4), the present catalyst exhibits superior TOF values in the hydroformylation of long-chain olefins. This enhanced activity originates from the CTF-TPA supports, which enable effective dispersion of active metallic species and capability to enrich olefin substrates within their hierarchical pore structures. These results highlight the unique advantages of CTF-TPA in constructing efficient heterogeneous catalytic systems for hydroformylation applications.

The catalytic performance of Rh/CTF-TPA in hydroformylation was further investigated across various olefin substrates to evaluate its activity, selectivity, and general applicability (Table 2). Long-chain olefins with different carbon chain lengths (C6, C8, C10, C12) and styrene were selected as substrates to assess their hydroformylation performance. Remarkably, Rh/CTF-TPA exhibited robust catalytic efficiency in all tested systems. Under optimized conditions (80 °C, 4 MPa syngas pressure, 1 h reaction time), both 1-hexene and 1-octene achieved over 80% conversion with favorable linear-to-branched ratios. Notably, Rh/CTF-TPA delivered 100% chemoselectivity in styrene hydroformylation. This originates from the highly conjugated structure of styrene that precludes isomerization reactions.

### 3.3. In Situ FT-IR

**Figure 6:**
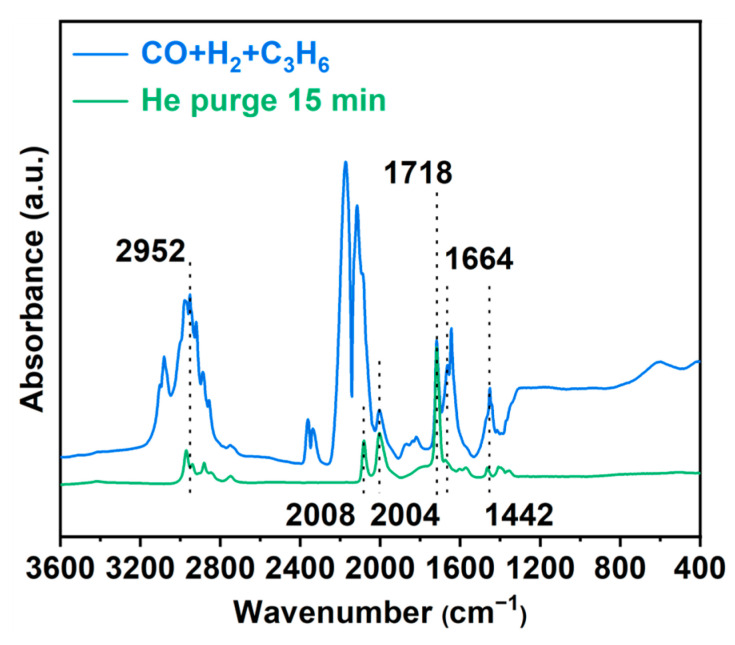
In situ FT-IR spectra of Rh/CTF-TPA upon reactant gas adsorption.

In situ Fourier transform infrared (FT-IR) spectroscopy revealed surface-adsorbed species on the catalyst during hydroformylation (as shown in Figure 6). After treating the catalyst with a syngas mixture (CO + H_2_ + C_2_H_6_) at 80 °C for 10 min followed by He purging, distinct absorption bands were identified: the peaks at 2952 cm^−1^ (C–H stretching), 1664 cm^−1^ (C = C stretching), and 1442 cm^−1^ (C–H scissoring) are assigned to adsorbed propylene [54]. The propylene molecules were stably adsorbed on the catalyst surface through π-bonding of the C = C double bond and weak interactions of the C–H bonds with Rh active sites. The incomplete desorption after He purging indicated strong adsorption, which may lead to the formation of surface intermediates. Two sharp peaks in the 2100–2200 cm^−1^ region, attributed to gaseous CO adsorption, gradually diminished and eventually disappeared after He purging. The bands at 2004 cm^−1^ and 2088 cm^−1^ are ascribed to Rh(CO)_2_ species, consistent with CO adsorption configurations, and the emergence of a peak at 1718 cm^−1^ corresponding to the C = O stretching vibration of an aldehyde group, coupled with the coexistence of Rh(CO)_2_ species, confirms that CO molecules insert into the Rh–alkyl bond to form aldehydes, aligning with the classical olefin–CO insertion mechanism of hydroformylation. These results indicate that, in addition to the atomically dispersed rhodium active sites, strong substrate adsorption may also be a contributing factor to the enhanced activity of the Rh/CTF-TPA catalyst in the hydroformylation reaction.

### 3.4. Catalyst–Substrate Adsorption

**Figure 7:**
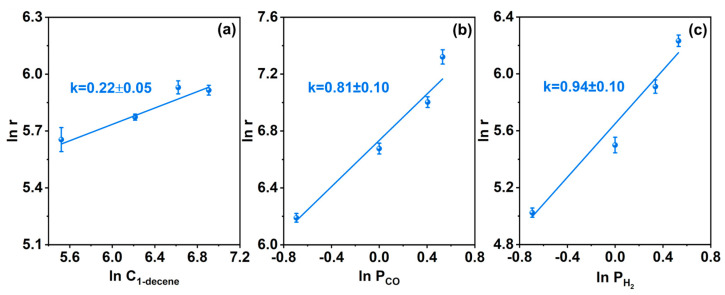
Kinetic reaction order to (**a**) 1-decene, (**b**) CO, and (**c**) H_2_ in the hydroformylation of 1-decene over Rh/CTF-TPA catalysts. The error bars in the figures show the differences between the three measurements.

The adsorption and activation of olefins play a critical role in the hydroformylation reaction mediated by supported Rh nanoparticle catalysts [55,56,57]. The kinetic characteristics of the 1-decene hydroformylation reaction catalyzed by the Rh/CTF-TPA catalyst were investigated. The experimental results indicated that the apparent reaction orders with respect to 1-decene, CO, and H_2_ were 0.22, 0.81, and 0.94 (Figure 7), respectively, which exhibited significant differences compared with those of the conventional hydroformylation systems. Compared with the positive reaction orders typically reported for supported Rh nanoparticles and homogeneous ligand-containing catalytic systems, the close-to-zero-order kinetic behavior of the olefin (0.22) reveals a unique surface reaction mechanism of this catalyst. The close-to-zero reaction order for the olefin indicates the insensitivity of the reaction rate to olefin concentration, which may be attributed to the enrichment of olefin molecules around Rh active sites in the Rh/CTF-TPA catalyst [58]. The high specific surface area and appropriate pore size distribution of the CTF-TPA support not only provide a favorable environment for the dispersion of Rh active sites but also facilitate the adsorption of olefin molecules.

**Figure 8:**
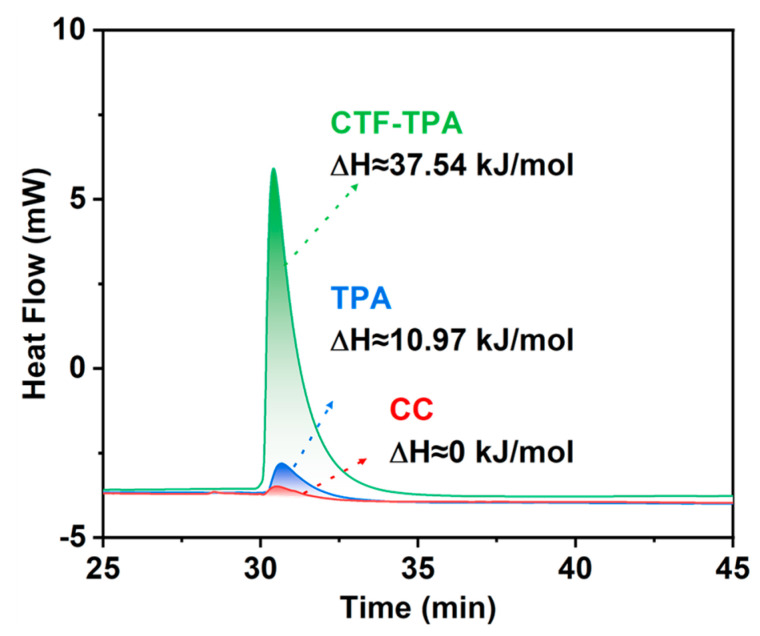
Calorimetric measurements of propylene adsorption at 298 K on the CTF-TPA support and its building blocks CC and TPA.

To gain deeper insights into the adsorption characteristics of catalyst surfaces toward olefins, propylene was employed as a probe molecule to quantitatively analyze the thermodynamic behavior of polymeric monomers (CC and TPA) and the CTF-TPA support using differential scanning calorimetry (DSC). As shown Figure 8, the results indicated that CC exhibited negligible adsorption behavior towards propylene. The adsorption enthalpy of TPA towards propylene was merely ∆H ≈ 10.97 kJ/mol, while the adsorption enthalpy of the CTF-TPA carrier, formed via polymerization, was significantly enhanced to ∆H ≈ 37.54 kJ/mol. This marked disparity in adsorption free energy underscores the critical role of the polymeric framework in facilitating specific interactions with olefin molecules. The thermodynamic data, in conjunction with the close-to-zero reaction kinetics for olefin concentration observed in previous kinetic studies, collectively confirm that the microstructure of the polymeric catalyst facilitates the formation of dynamic olefin-enriched zones near active sites, thereby enhancing hydroformylation activity.

### 3.5. Mechanism of Olefin Hydroformylation over Rh/CTF-TPA

**Figure 9:**
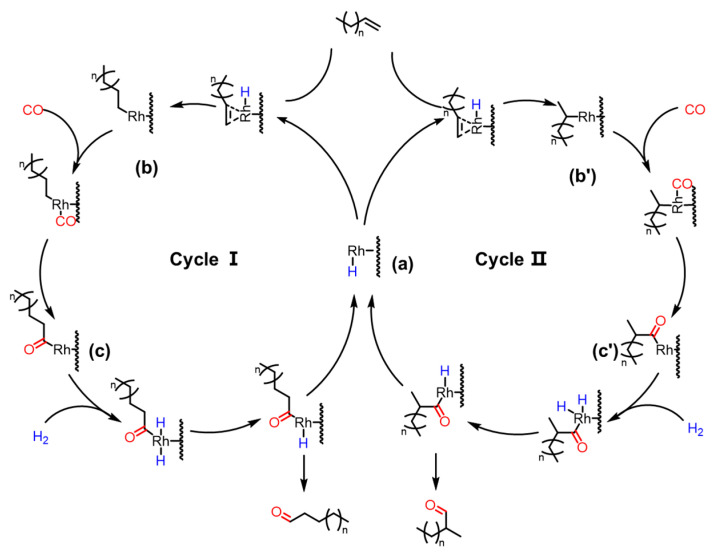
The reaction pathway of olefin hydroformylation to aldehydes on Rh/CTF-TPA, where (**a**) represents the [RhH(CO)xLy] active center; (**b**,**b′**) represent alkyl–rhodium intermediates; (**c**,**c′**) represent acyl–rhodium species.

In the 1960s, Heck and Breslow proposed the pioneering reaction mechanism for hydroformylation based on Co-based catalytic systems, which has since become the widely accepted theoretical framework in this field. This mechanistic pathway has been crucially extended to Rh-catalyzed hydroformylation reactions. Inspired by homogeneous reaction mechanisms, a plausible reaction mechanism for the Rh/CTF-TPA-catalyzed hydroformylation of olefins has been proposed, as illustrated in Figure 9. In the Rh/CTF-TPA catalytic system, nitrogen sites on the CTF-TPA support anchor Rh species to form the [RhH(CO)_x_L_y_] active center (a). Subsequently, olefin coordinates to the Rh center, and the double bond inserts into the H-Rh bond to generate an alkyl–rhodium intermediate (b and b′). The regioselectivity of olefin insertion (terminal vs. internal insertion) determines the formation of linear or branched aldehydes (Cycle I and II, respectively). CO then inserts into the Rh-C bond to yield an acyl–rhodium species (c and c′), which undergoes reductive elimination with H_2_ to release the aldehyde product and regenerate the initial active catalyst.

## 4. Conclusions

In summary, we have successfully developed a stable nitrogen-rich CTF material through a Friedel–Crafts alkylation synthetic strategy. Utilizing this CTF as a support, a novel rhodium-based heterogeneous catalyst (Rh/CTF-TPA) was rationally designed for the hydroformylation of long-chain olefins. Under optimized conditions, the Rh/CTF-TPA catalyst exhibited remarkable catalytic performance in 1-decene hydroformylation, achieving a conversion of 89.6% and aldehyde selectivity of 84.4%. Comprehensive experimental studies and characterization demonstrate that the abundant nitrogen-coordinating sites and unique porous architecture of CTF materials not only enable atomic-scale dispersion of rhodium species but also induce pronounced reactant enrichment within confined nanochannels, thereby enhancing local substrate concentration. These mechanisms collectively contribute to enhanced reaction kinetics and catalytic efficiency in the hydroformylation process. The integration of spatial confinement and coordination stabilization mechanisms in this work establishes a new structural design paradigm for designing heterogeneous catalysts that reconcile high activity and selectivity. The insights gained from this work provide a versatile platform for optimizing catalytic processes in fine chemical synthesis and industrial applications.

## Figures and Tables

**Table 1:** Comparison of Rh/CTF-TPA and homogeneous Rh(CO)_2_(acac) in hydroformylation ^1^.

Catalyst	Conv. (%)	Sel. (%)	L/B	TOF (h^−1^) ^2^
Rh(CO)_2_(acac)	26.9	48.8	1.83	925
Rh/CTF-TPA	57.5	55.8	2.51	1929

^1^ Reaction conditions: Rh: substrate = 1: 4500, toluene (4 mL), CO:H_2_ = 1:1 (4 MPa), at 80 °C for 1 h. The products were analyzed by GC and GC–MS. ^2^ $TOF=\frac{n_{substrate}}{n_{Rh}\times t}$, where n_substrate_ is the moles of converted substrate (mol), n_Rh_ represents the total moles of Rh (mol): $n_{Rh}=\frac{Rh\;loading\left(wt\%\right)\times Catalyst\;mass\left(g\right)}{M_{Rh}}$, t denotes the reaction time (h).

**Table 2:** Hydroformylation of different olefins catalyzed by Rh/CTF-TPA ^1^.

Catalyst	Conv. (%)	Sel. (%)	L/B	TOF (h^−1^) ^2^
1-hexene	85.9	85.4	2.47	3187
1-octene	88.9	72.0	2.47	2960
1-decene	57.5	84.3	2.51	1929
1-dodecene	58.3	80.2	2.50	1992
styrene	60.1	100	0.70	2033

^1^ Reaction conditions: catalyst (15 mg), substrate (5 mmol), toluene (4 mL), CO:H₂ = 1:1 (4 MPa), at 80 °C for 1 h. The products were analyzed by GC and GC–MS. ^2^ $TOF=\frac{n_{substrate}}{n_{Rh}\times t}$, where n_substrate_ is the moles of converted substrate (mol), n_Rh_ represents the total moles of Rh (mol): $n_{Rh}=\frac{Rh\;loading\left(wt\%\right)\times Catalyst\;mass\left(g\right)}{M_{Rh}}$, t denotes the reaction time (h).

## Data Availability

The original contributions presented in this study are included in the article/Supplementary Material. Further inquiries can be directed to the corresponding authors.

## Supplementary Materials

The following supporting information can be downloaded at: https://www.mdpi.com/article/10.3390/ma18122691/s1. Figure S1. Photographs of the synthesized catalysts: (a) CTF-TPA and (b) Rh/CTF-TPA; Figure S2: XRD pattern of CTF-TPA and Rh/CTF-TPA; Figure S3: SEM images of (a,b) CTF-TPA and (c,d) Rh/CTF-TPA; Figure S4: (a,b) TEM and (c,d) HAADF-STEM images of Rh/CTF-TPA, together with EDX analysis (e); Figure S5: (a) N_2_ adsorption/desorption isotherm and (b) pore size distribution of CTF-TPA and Rh/CTF-TPA; Table S1: Specific surface area and pore structure parameters of CTF-TPA and Rh/CTF-TPA; Figure S6: Thermogravimetric curves of CC, TPA, CTF-TPA, and Rh/CTF-TPA; Figure S7: XPS spectra of (a) Rh 3d of Rh(CO)_2_(acac); (b) N 1s of CTF-TPA; Table S2: Optimization of reaction conditions for 1-decene hydroformylation with Rh/CTF-TPA; Table S3: Effect of solvent on the catalytic activity of Rh/CTF-TPA in hydroformylation of 1-decene; Table S4. The comparison of long-chain olefins hydroformylation activities of reported Co-based and Rh-based heterogeneous catalytic systems.
