# Supplementary material for: Boosting Hydroformylation via Reactant Enrichment in Covalent Triazine Frameworks with Atomically Dispersed Rh
Source: Materials (Basel). 2025 Jun 7;18(12):2691. doi: 10.3390/ma18122691 (PMC12193888; doi:10.3390/ma18122691)
Supplement: Supplementary file 1 [file materials-18-02691-s001.zip › materials-3629926-supplementary.pdf]

materials

Supplementary

Boosting Hydroformylation via Reactant Enrichment in Covalent Triazine Frameworks with atomically dispersed Rh

Xinguo Li [1†], Xiangjie Zhang [2†], Gaolei Qin [2], Peng He[2,*] and Yajuan Hao[1,*]

[1] School of Chemistry and Chemical Engineering, Shanxi University, Taiyuan 030006, China; lixinguo0105@163.com
[2] State Key Laboratory of Coal Conversion, Institute of Coal Chemistry, Chinese Academy of Sciences, Taiyuan 030001, China; zhangxiangjie18@mails.ucas.ac.cn (X.Z.); qingaolei21@mails.ucas.ac.cn (G.Q.)
* Correspondence: hepeng@sxicc.ac.cn (P. H.); yjhao@sxu.edu.cn (Y. H.)
† These authors contributed equally to this work.

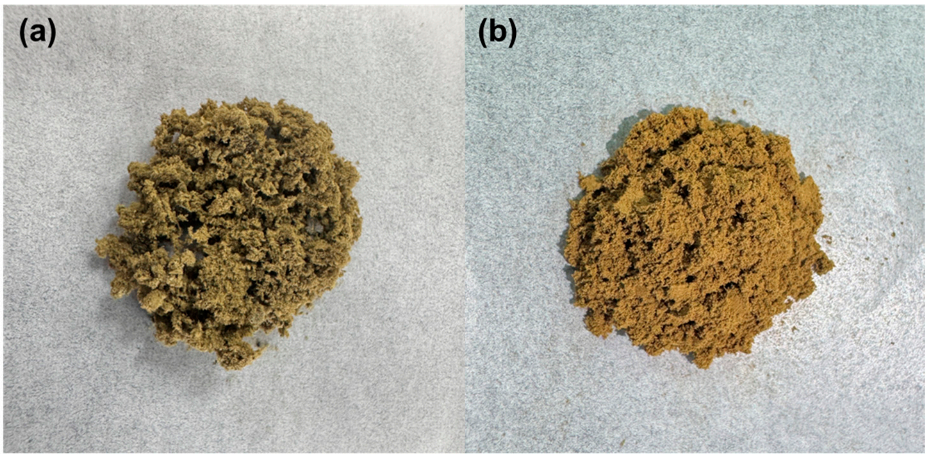

Figure S1. Photographs of the synthesized catalysts: (a) CTF-TPA and (b) Rh/CTF-TPA.

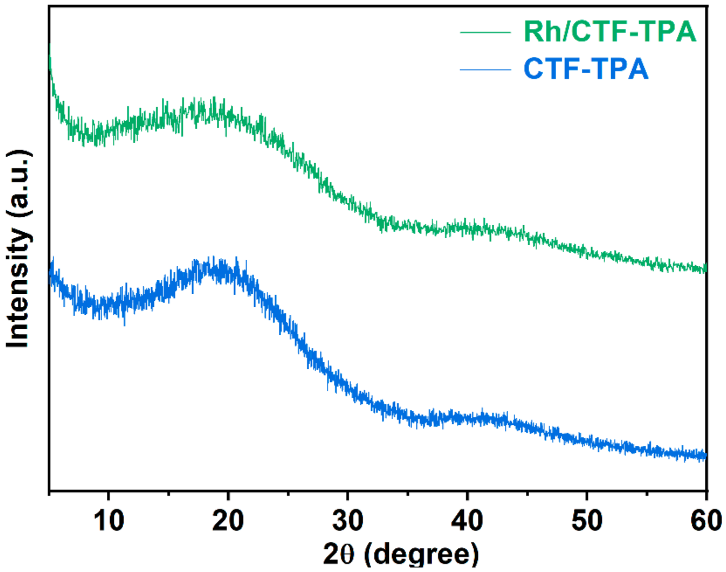

Figure S2. XRD pattern of CTF-TPA and Rh/CTF-TPA.

 https://doi.org/10.3390/ma18122691

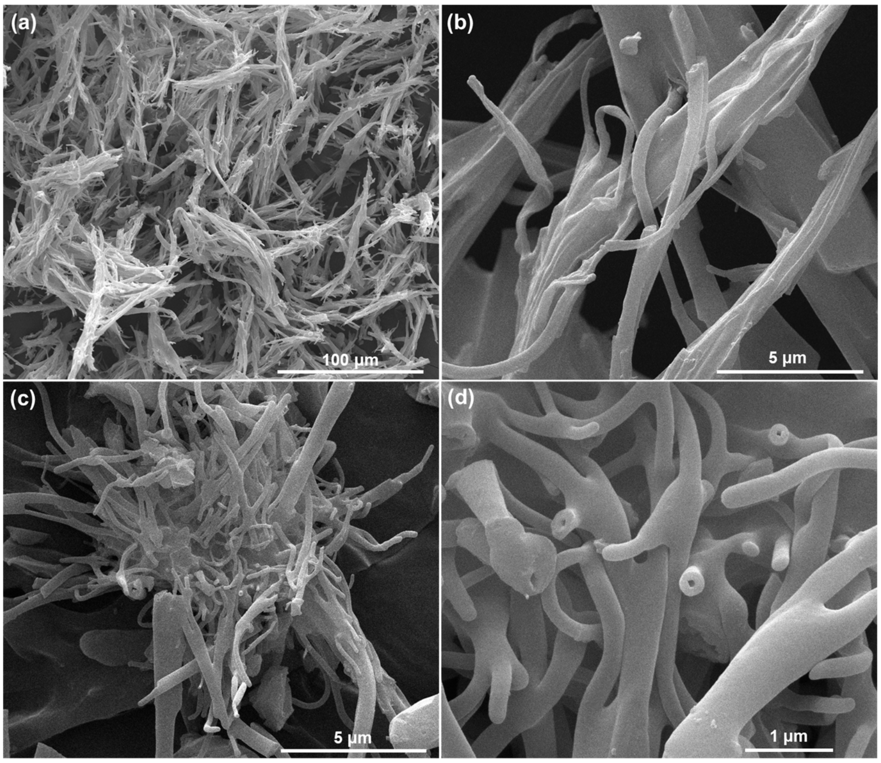

Figure S3. SEM images of (a, b) CTF-TPA and (c, d) Rh/CTF-TPA.

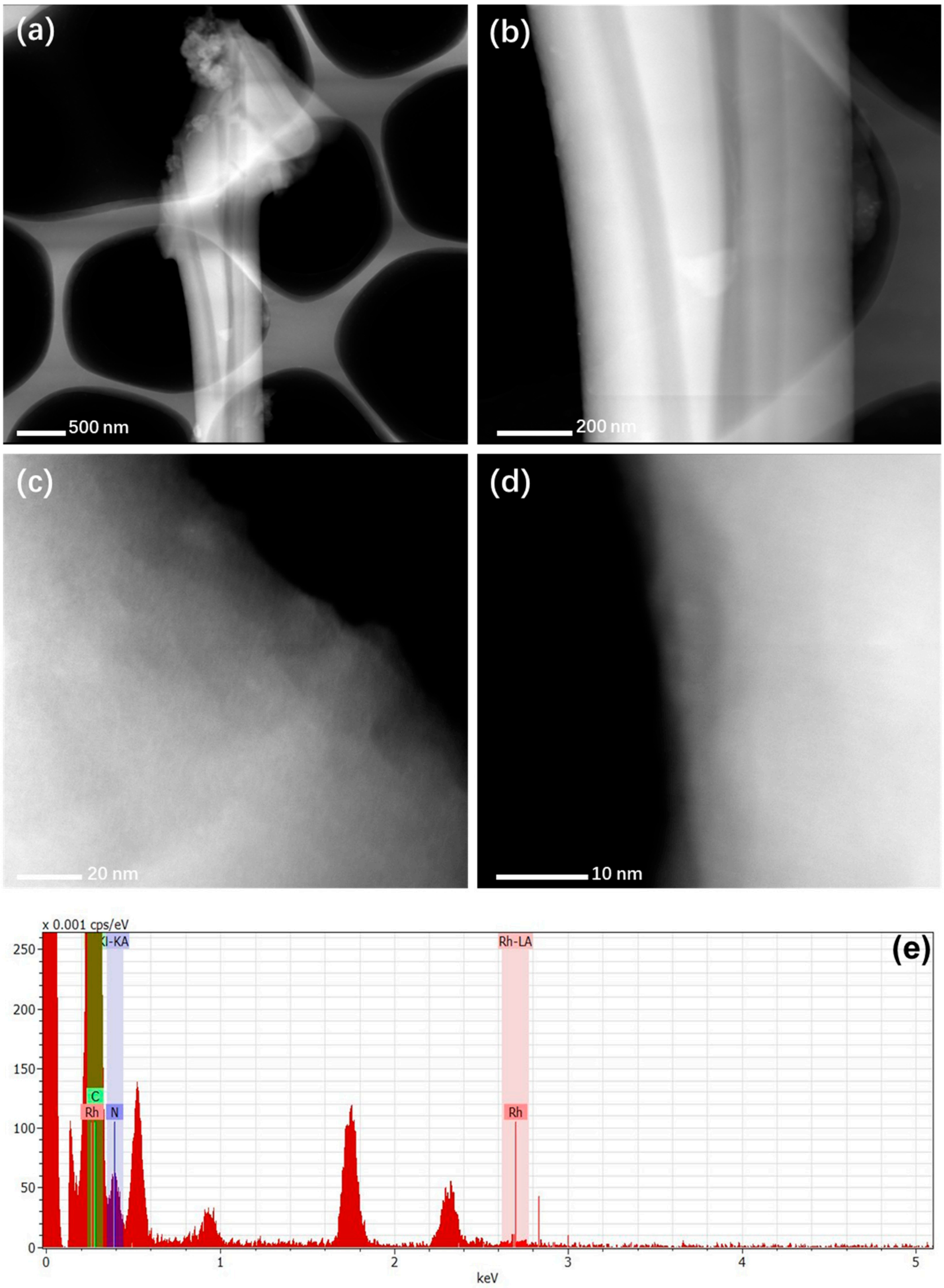

Figure S4. (a) and (b) TEM, (c) and (d) HAADF-STEM images of Rh/CTF-TPA, together with EDX analysis(e).

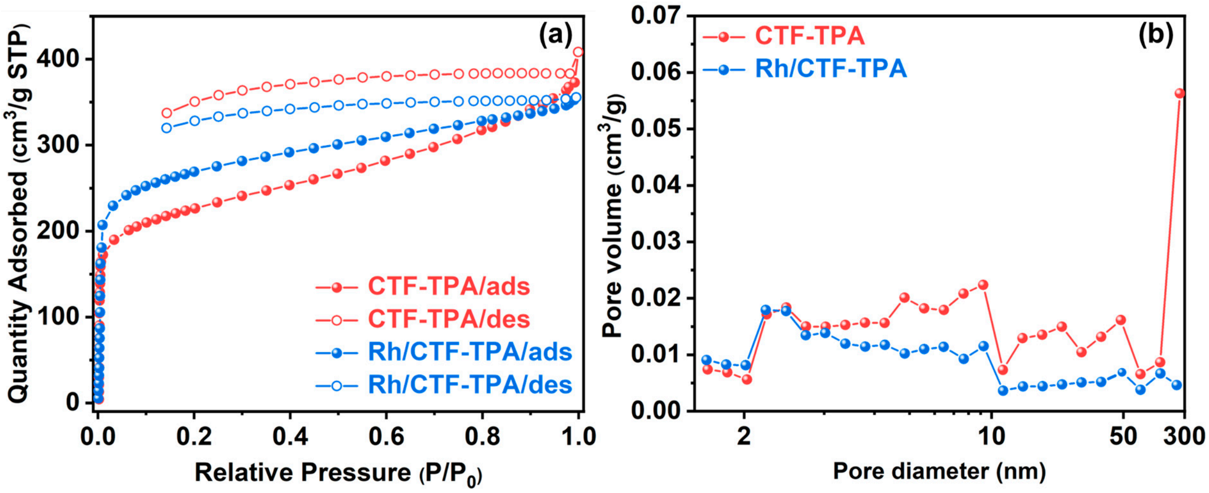

Figure S5. (a) N_2 adsorption/desorption isotherm and (b) pore size distribution of CTF-TPA and Rh/CTF-TPA.

Table S1. Specific surface area and pore structure parameters of CTF-TPA and Rh/CTF-TPA.

Sample	S_{BET} ($m^2 g^{-1}$)	S_{Ext} ($m^2 g^{-1}$)	Vp(total) ($cm^3 g^{-1}$)	Vp(micro) ($cm^3 g^{-1}$)	Pore Size (nm)
CTF-TPA	893.6	368.9	0.48	0.25	5.65
Rh/CTF-TPA	816.0	335.5	0.65	0.23	3.89

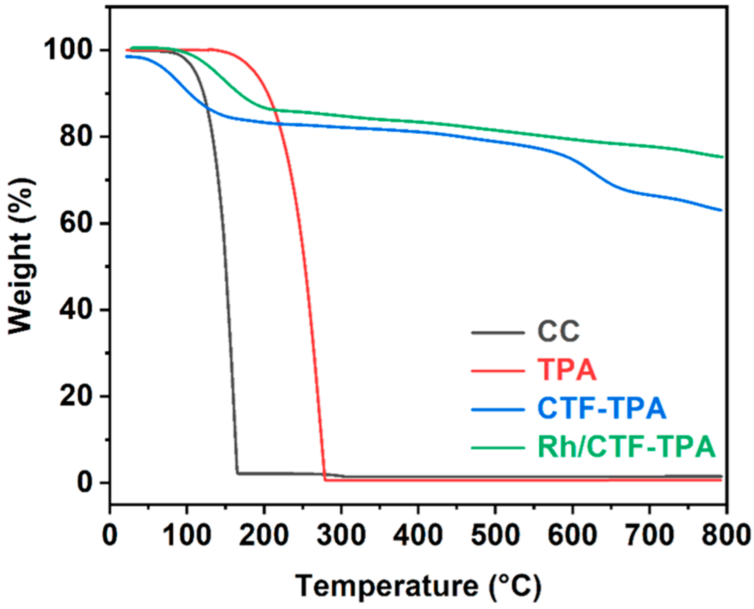

Figure S6. Thermogravimetric curves of CC, TPA, CTF-TPA, Rh/CTF-TPA.

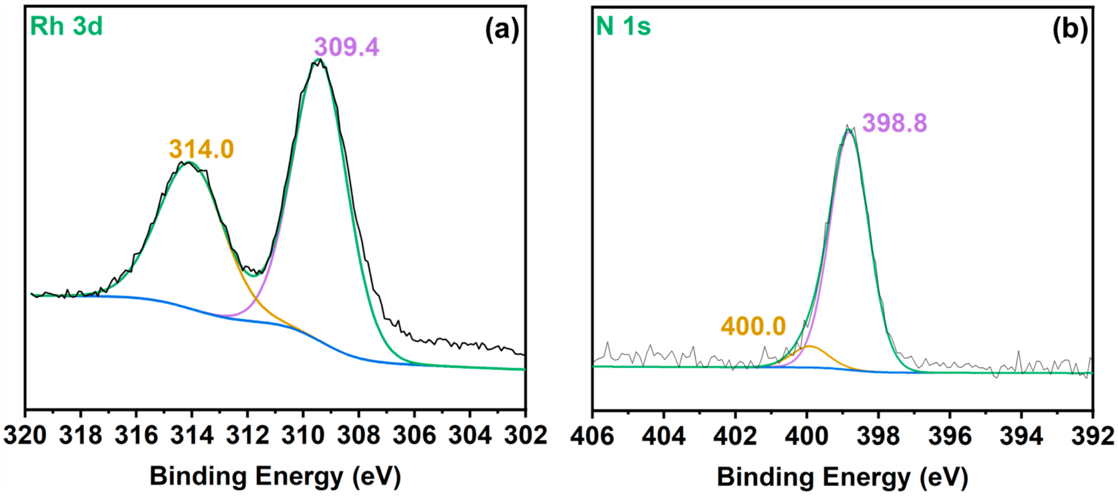

Figure S7. XPS spectra of (a) Rh 3d of $Rh(CO)_2(acac)$; (b) N 1s of CTF-TPA.

Table S2. Optimization of reaction conditions for 1-decene hydroformylation with Rh/CTF-TPA [1].

Entry	T(°C)	P(MPa)	Conv.(%)	Sel.(%) [2]	L/B [3]
1	70	4	43.6	90.4	2.07
2	80	4	89.3	87.1	2.51
3	90	4	91.6	79.1	1.42
4	100	4	95.0	74.9	1.19
5	80	1	37.4	69.3	2.81
6	80	2	55.7	72.2	2.62
7	80	3	72.9	83.0	2.47
8	80	5	88.6	91.1	2.11

[1] Reaction conditions: catalyst (15 mg), 1-decene (5 mmol), toluene (4 mL), reaction time (2 h), $CO:H_2 = 1:1$. The products were analyzed by GC and GC-MS; [2] Selectivity for undecanal; [3] Ratio of linear to branched aldehydes.

Table S3. Effect of solvent on the catalytic activity of Rh/CTF-TPA in hydroformylation of 1-decene [1].

Entry	Solvent	Conv.(%)	Sel.(%) [2]	L/B [3]
1	Toluene	89.6	84.4	2.51
2	1,4-Dioxane	84.2	82.7	1.21
3	Ethyl acetate	87.3	83.4	1.30
4	Acetone	86.4	77.8	1.39
5	Cyclohexane	88.5	86.8	2.31
6	DMF	49.2	43.5	2.81

[1] Reaction conditions: catalyst (15 mg), 1-decene (5 mmol), solvent (4 mL), $CO:H_2$ = 1:1 (4 MPa), at 80 °C for 2 h. The products were analyzed by GC and GC-MS.[2] Selectivity for undecanal; [3] Ratio of linear to branched aldehydes.

Table S4. The comparison of long-chain olefins hydroformylation activities of reported Co-based and Rh-based heterogeneous catalytic systems.

Entry	Catalyst	Substrate	Conditions	TOF(h^{-1})	Ref.
1	$Co_2(CO)_8$/ MixUMCM-1-NH_2	1-hexene	100°C, 3MPa	18	[47]
2	$Co_2(CO)_8$/ MOF-74(Zn)	1-hexene	100°C, 3 MPa	12	[47]
3	Co-B/TNTs	1-octene	100°C, 6 MPa	2.1	[48]
4	Co/POL-$POPh_3$	2-octene	150°C, 3 MPa	69	[49]
5	RhZn/SBA-15	1-octene	100°C, 3 MPa	1134	[50]
6	Rh/TImT-TBPT	1-octene	100°C, 6 MPa	216	[51]
7	EG-Rh/rGO	1-octene	90°C, 5MPa,	628	[52]
8	DMSN-C18N-0.8	1-decene	100°C, 2 MPa	250	[53]
9	Rh/CTF-TPA	1-decene	80°C, 4 MPa	1929	This work
